# Mr. Richard Walker in Reply to Mr. Woodham

**Published:** 1816-10

**Authors:** 


*For the London Medical and Physical Journal.*

### Mr. Richard Walker *in Reply to Mr. Woodham.*

IN looking over your Journal for August, I unexpectedly found a second attack upon me by Mr. Woodham.

I must confess, from the conduct and termination of the former controversy, I was in hopes I should never again have been troubled in like manner from the same quarter.

As

As it is my determination in this instance not to enter into any controversy respecting the merits or demerits of my various papers on small-pox and vaccination, having delivered my opinions thereon in the most explicit, intelligible, and conscientious manner, according to my experience and judgment, I shall forbear entirely commenting upon the remarks of Mr. W.

Those who remember, or are disposed to revert to a former controversy, alluded to above, between Mr. W. and myself, will not, I think, be inclined to attribute this determination to any apprehension on my part, respecting the result, were I to enter into it.

I shall avail myself, however, of this opportunity to express my consciousness that several of my communications may have been indebted, for their introduction to the public, more to the courtesy of the editors than to their intrinsic value; yet, I am unwilling to believe, that the editors would be so extremely accommodating as to sacrifice entirely their judgment to me, or to any one else.

In return for Mr. W.'s *kind* remarks and admonitions towards *me*, I shall take the liberty of offering *him* a little friendly and salutary advice, as I consider it, viz. that in his future remarks upon the productions of others, should he feel disposed to amuse himself that way, he would incline less to *asperity*, and more towards *urbanity*, than he has towards me; and, likewise, to recollect, that the progress of science is better promoted, and its cause better advocated, by a concise plain style of language, than by *verbosity* and *pedantry*.

With respect to myself, I beg leave to apprize Mr. W., that, should he, at any future time, be disposed to comment upon any communication, he must not be surprised if I should neglect to notice it; and request he will attribute this omission to other motives than a consciousness of my incompetency to answer it; viz. to want of leizure, or want of excitement.

You will have the goodness, gentlemen, to place *this* unprofitable trespass upon the valuable sheets of your Journal to the account of *Mr. Woodham*.

*Oxford;*
*August* 8, 1816.

